# High-throughput microbial culturomics using automation and machine learning

**DOI:** 10.1038/s41587-023-01674-2

**Published:** 2023-02-20

**Authors:** Yiming Huang, Ravi U. Sheth, Shijie Zhao, Lucas A. Cohen, Kendall Dabaghi, Thomas Moody, Yiwei Sun, Deirdre Ricaurte, Miles Richardson, Florencia Velez-Cortes, Tomasz Blazejewski, Andrew Kaufman, Carlotta Ronda, Harris H. Wang

**Affiliations:** 1 Department of Systems Biology, Columbia University, New York, NY USA; 2 Department of Biomedical Informatics, Columbia University, New York, NY USA; 3 Department of Pathology and Cell Biology, Columbia University, New York, NY USA

**Keywords:** Microbiology techniques, Microbiome, Bacterial techniques and applications

## Abstract

Pure bacterial cultures remain essential for detailed experimental and mechanistic studies in microbiome research, and traditional methods to isolate individual bacteria from complex microbial ecosystems are labor-intensive, difficult-to-scale and lack phenotype–genotype integration. Here we describe an open-source high-throughput robotic strain isolation platform for the rapid generation of isolates on demand. We develop a machine learning approach that leverages colony morphology and genomic data to maximize the diversity of microbes isolated and enable targeted picking of specific genera. Application of this platform on fecal samples from 20 humans yields personalized gut microbiome biobanks totaling 26,997 isolates that represented >80% of all abundant taxa. Spatial analysis on >100,000 visually captured colonies reveals cogrowth patterns between *Ruminococcaceae*, *Bacteroidaceae*, *Coriobacteriaceae* and *Bifidobacteriaceae* families that suggest important microbial interactions. Comparative analysis of 1,197 high-quality genomes from these biobanks shows interesting intra- and interpersonal strain evolution, selection and horizontal gene transfer. This culturomics framework should empower new research efforts to systematize the collection and quantitative analysis of imaging-based phenotypes with high-resolution genomics data for many emerging microbiome studies.

## Abstract (web summary)

A machine learning isolation and genotyping platform enable high-throughput bacterial culture generation.

## Main

Metagenomics offers the ability to broadly survey the composition of diverse microbial ecosystems ranging from soil communities to the gut microbiome. Yet microbes need to be isolated and cultured to mechanistically dissect their functional roles in habitat and the myriad of interspecies processes that occur. Traditional cultivation methods relying on ‘brute force’ random colony picking are tedious and labor-intensive^1–4^. Serial dilution-based isolation methods using 96- or 384 wells are resource-intensive and result in repeated isolation of the same dominant strains from the population^5^. Microfluidic systems enable growth in nanoliter reactors, but clonal isolates are difficult to extract^6,7^. Given that a typical microbiome can contain hundreds to thousands of unique species exhibiting a long-tailed abundance distribution^8^ (that is, few dominate while most are rare), generating comprehensive strain collections via systematic culturomics remains an important and outstanding challenge.

Microbes can be distinguished based on their diverse phenotypes, whether by their ability to grow in certain media or the metabolites they produce^9–12^. Growth-based selection can enhance the isolation of rare species, for example, with growth media containing different nutrients or antibiotics^1,2,13^. Mass spectrometry spectra can be used to differentiate between species^14,15^, but the approach is low-throughput and requires manual processing. Imaging-activated cell sorting has been developed to isolate eukaryotic cells based on multidimensional images, but this method requires sophisticated instrumentation and has not been implemented for bacteria^16^. With recent advances in artificial intelligence (AI) and deep learning models trained to discern nuanced features in multidimensional imaging and biological data^17^, machine learning (ML) of combined phenotypic and genomic data streams is poised to transform next-generation microbial culturomics.

Here we describe an ML-guided robotic strain isolation and genotyping platform that enables rapid and high-throughput generation of cultured biobanks on demand. This system uses an intelligent imaging-based algorithm to increase the taxonomic diversity of culturomics compared to a random-picking method. We demonstrated the utility of this system by anaerobically generating personalized isolate biobanks for 20 human participants, yielding a total of 26,997 isolates with 1,197 high-quality draft genomes, spanning 394 16S amplicon sequence variants (ASVs). Using the paired genomic and morphological information for each isolate, we trained an ML model that can predict taxonomic identity based only on colony morphology. Application of this ML model led to an improvement in targeted isolation of microbes of interest. Large-scale imaging analysis of all colonies grown on agar plates revealed interesting species-specific growth patterns and interspecies interactions. Whole-genome analysis from personalized biobanks uncovered person-specific strain-level variation and signatures of horizontal gene transfer (HGT) within major gut phyla. We further developed an open-access web-based database (http://microbial-culturomics.com/) containing searchable genotypic, morphologic and phenotypic data of all isolates generated by automated culturomics as a unique and expanding community resource for the microbiome field.

## Results

### Data-driven culturomics using phenotypes and automation

Colony picking is a classic microbiology method for clonally isolating bacterial strains. Colony growth on plates depends on many factors, including the composition of the media (for example, available nutrients), atmospheric conditions (for example, level of oxygenation), presence of inhibitory molecules (for example, antibiotics), pH, humidity and effects of other diffusible metabolites derived from nearby colonies^18–20^. Different colony morphologies are observed based on strain-specific physiological differences, influenced by cell shape, rigidity, motility and growth kinetics, as well as production of pigmented molecules or extracellular matrices and surfactants^9–12^. Although these colony traits are readily quantifiable, they are rarely documented during colony isolation. As a result, selective colony picking using visual features is generally qualitative and not standardized, and outcomes can vary substantially between experiments and experimentalists. To address these shortcomings, we devised a platform dubbed Culturomics by Automated Microbiome Imaging and Isolation (CAMII) to systematize culturomics with both morphologic and genotypic data for colony isolation and functional analysis.

**Fig. 1:**
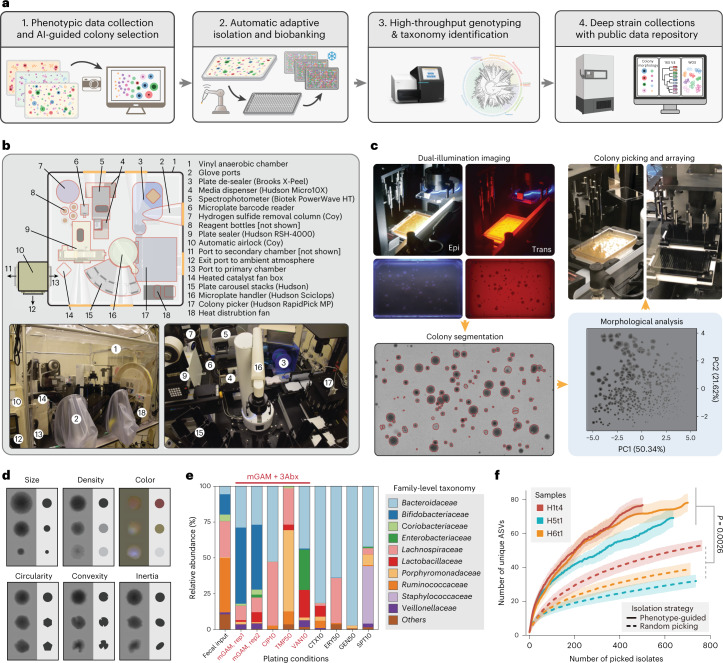
A data-driven microbial isolation strategy using phenotypic and morphologic features. **a**, Framework of phenotype and morphology-driven strain isolation and data collection of the human gut microbiome. Human fecal samples were plated and cultured under different antibiotics selection and morphologically diverse colonies were then isolated, biobanked and analyzed by downstream sequencing. **b**, Setup of the automated anaerobic microbial isolation and cultivation system CAMII. **c**, Illustration of morphology-guided colony isolation on CAMII. Colonies grown on plates are imaged under trans- and epi-illumination and subjected to contour segmentation and morphologic features extraction. Data are analyzed by PCA to identify the set of most morphologically diverse colonies that are then isolated by an integrated colony picker. **d**, Illustration of diverse colony morphology on plates. Colony size and shape features were extracted from transilluminated images, and colony color features were extracted from epi-illuminated images. **e**, Fecal sample H1t1 were cultured with seven different antibiotics to evaluate their capacity to yield the most unique and diverse bacteria by 16S analysis at the family level. Ciprofloxacin, trimethoprim and vancomycin were selected for subsequent colony isolations. **f**, Number of unique ASVs obtained from phenotype-guided isolation compared to random isolation of three human fecal samples H1t4, H5t1 and H6t1. Isolation was performed by CAMII; random isolation was performed on a random subset of all detected colonies on the plates, and phenotype-guided isolation was performed on morphology-selected colonies by the algorithm (Supplementary Fig. 1b). *P* value is calculated by a two-sided paired *t*-test on area under the curve. Ribbons on the curves represent the standard deviations of the number of obtained unique ASVs by the algorithm.

The CAMII platform consists of four key elements (Fig. 1a) discussed as follows: (1) an imaging system that collects morphology data of colonies and an AI-guided colony selection algorithm, (2) an automated colony-picking robot for high-throughput isolation and arraying of isolates, (3) a cost-effective pipeline to rapidly generate genomic data for picked isolates and (4) a physical isolate biobank and digital database with searchable colony morphology, phenotype and genotype information. Thus, this end-to-end culturomics platform can produce isolate collections from diverse input microbiomes with minimized manual labor. The entire imaging and isolation system is built using off-the-shelf components housed in an anaerobic chamber that provides real-time control of temperature, humidity and oxygen levels (Fig. 1b and Supplementary Table 1). The CAMII robot has an isolation throughput of 2,000 colonies per hour and can handle 12,000 colonies per run, which is >20 times higher capacity and faster than manual colony isolation by a person. To ensure that our genomic analysis capacity matches the robotic isolation throughput, we also developed a low-cost, high-throughput sequencing pipeline that leverages liquid handling automation to generate barcoded libraries for 16S rRNA sequencing or whole-genome sequencing (WGS; Methods). The cost per isolate in this pipeline is $0.45 for colony isolation and genomic DNA (gDNA) preparation, $0.46 for 16S rRNA sequencing and $6.37 for WGS at a coverage of >60× on an Illumina HiSeq platform, which is substantially cheaper than commercial services (Supplementary Table 2).

A key unique feature of the CAMII platform is the imaging system that collects and learns from morphological data of bacterial colonies (Fig. 1c). Specifically, transilluminated images, which show height, radius, and circularity of a colony and epi-illuminated images, which show color and complex morphological features such as wrinkling, are captured on CAMII to yield a multidimensional and quantifiable morphological dataset. We developed a custom colony analysis pipeline that segments colonies along diverse morphological features (Methods; Supplementary Table 3 and Supplementary Fig. 1). Area, perimeter and mean radius reflect colony size, while circularity, convexity and inertia reveal colony shape. Pixel intensities and their variances in the red, green and blue (RGB) channels highlight any density gradations and colors across a colony (Fig. 1d). We next reasoned that morphologically distinct colonies are more likely to be phylogenetically diverse, which could be used to improve colony isolation. Thus, we developed an imaging-guided ‘smart picking’ strategy to isolate more diverse isolates by embedding colonies in a multidimensional Euclidean space based on captured features and selecting maximally distant points in this space representing the most morphologically distinct colonies (Supplementary Fig. 1; Methods). To further increase the diversity of bacteria that can be cultured and examined, CAMII also uses different antibiotic supplements to enrich the most unique and diverse subsets of microbes^1,13^ (Supplementary Fig. 2a,b). For instance, in a healthy human gut microbiome sample (H1t1), three antibiotics (ciprofloxacin, Cip; trimethoprim, Tmp; vancomycin, Van) with different mechanisms of action elicited the most distinct enrichment cultures (Fig. 1e and Supplementary Fig. 2c).

To systematically evaluate the capacity and fidelity of imaging-guided colony isolation, we applied CAMII to gut microbiome samples from three human volunteers (H1t4, H5t1 and H6t1; Supplementary Table 4). Morphological data from plated colonies were analyzed by principal component analysis (PCA) to assess the most informative visual features (Fig. 1c and Supplementary Fig. 1c; Methods). Interestingly, colony density and size were the most dominant signatures (principal components 1 and 2, respectively) that together accounted for 72.0% of the morphological variance (Supplementary Fig. 3). We then used the CAMII robot to isolate 6,144 colonies, roughly half of them were randomly picked from mGAM plates and another half by using our imaging-guided ‘smart picking’ strategy and antibiotic selection. Isolates were grown in 384 wells and subjected to 16S rRNA sequencing for taxonomy identification. Unique 16S V4 sequences were then clustered into ASVs (100% identity cutoff) that provide approximate species-level identity^21^. Remarkably, colony isolation informed by phenotypic data yielded a substantially more diverse set of ASVs than compared to random isolation for all three microbiome samples (Fig. 1f). For example, to obtain 30 unique ASVs, we require only 85 ± 11 colonies to be isolated using our imaging selective strategy compared to 410 ± 218 colonies needed by random selection. Notably, this enhanced isolation efficiency was maintained throughout picking, implying that there is a sustained advantage in using our strategy at a range of desired isolation depth (Supplementary Fig. 4a), and the generated isolate collection better represented the underlying input microbial diversity and was substantially more even in composition as measured by Shannon’s equitability (Supplementary Fig. 4b). Phylogenetic analysis of isolates showed that CAMII-optimized colony picking substantially improved the diversity of obtained microbes (Supplementary Fig. 5). This advantage is particularly evident given that finding unique ASVs becomes asymptotically more difficult with an increasing number of isolates. Altogether, these results demonstrated our AI-guided data-driven isolation framework in the CAMII platform can substantially increase the efficiency of culturomics and lessened the labor to isolate especially rare species.

### Rapid generation of personalized gut isolate biobanks

**Fig. 2:**
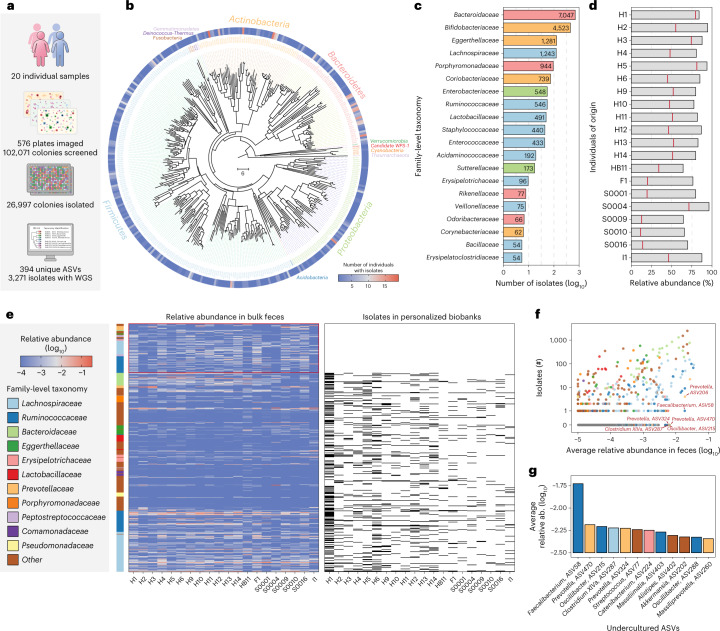
Generation of personalized gut isolate biobanks for 20 individuals. **a**, Statistics of 20 personalized gut isolate biobanks. **b**, Phylogenetic tree of 394 ASVs covered by 26,997 gut microbiome isolates in this study. Neighbor-joining tree of phylogeny was constructed based on 16S V4 sequences. Branch color distinguishes bacterial phylum, and the outer circle shows the prevalence of isolated ASVs in the 20 biobanks. **c**, Number of isolates for top 20 family-level taxonomy. **d**, Accumulated relative abundance of the ASVs represented by isolates from personalized biobanks in original fecal samples. The bars show isolates from any individual in the entire collection and the red lines show isolates derived from the same person. **e**, Heatmaps for relative abundance of abundant ASVs in original fecal samples and presence or absence in the biobanks. ASVs with average relative abundance > 0.1% are shown and the side bar on the left represents their family-level taxonomy. ASVs found in personalized biobanks are shown as black bars in the right heatmap and uncultured ASVs not found in any biobank are highlighted. **f**, Correlation of average relative abundance in original feces sample and number of isolates in entire collection for ASVs. Highly abundant ASVs that are difficult to culture, that is, with fewer isolates, are highlighted. **g**, Average relative abundance of top abundant ASVs but with no more than 2 isolates in the entire collection. Color of bars represents family-level taxonomy.

While microbiomes from different people may share similar sets of bacterial species, the strains belonging to these species are highly unique to the individual and may co-colonize the same host for many years^22,23^. We sought to showcase the utility of CAMII to generate personalized gut isolate collections for 20 healthy people (Supplementary Table 4 and Supplementary Fig. 6a,b). A total of 102,071 colonies were visually analyzed and 26,997 colonies were picked and taxonomically identified by 16S rRNA sequencing (Fig. 2a), yielding 394 unique ASVs that cover a broad diversity of healthy commensal gut microbiome (Fig. 2b,c and Supplementary Table 5).

To assess the comprehensiveness of this isolate collection, we calculated the abundance of isolated ASVs in the corresponding fecal samples by bulk 16S rRNA sequencing (Fig. 2d). Remarkably, for each individual, 80.9 ± 9.4% of the ASVs by abundance are represented at least once in the entire isolate collection. Isolates derived from each person constituted on average 45.6 ± 21.6% of the total bacterial ASV abundance within that individual (Fig. 2d). Moreover, comparison of isolate collections and bulk feces samples showed most of the highly abundant and prevalent ASVs are isolated at least once in the collection (Supplementary Fig. 6c–e). Moreover, each personalized isolate collection mimics the bulk feces sample with comparable microbiome profiles and Shannon’s diversity index (Supplementary Fig. 6f,g).

In all, we demonstrated the use of CAMII to build a deep human gut isolate collection containing 26,997 isolates spanning 394 ASVs, with a rich set of linked morphologic, phenotypic, taxonomic and WGS data. To increase its utility for the research community, we further developed a searchable online resource (http://microbial-culturomics.com) to house all CAMII-enabled biobank data including genomes, phenotypes and images. We envision this portal will facilitate further genotype-to-phenotype analyses and lead to more shared isolate collections from other environments.

### Identifying undercultured ‘dark matter’ gut microbiome

Previous studies have observed that many microbes from different environments are difficult to culture in the laboratory^24,25^. We therefore leveraged our systematically generated isolate biobanks to assess the culturability of the human gut microbiome and to identify bacterial ASVs that remain recalcitrant to isolation in our experimental setting. Across all 20 personalized isolate collections, we determined whether abundant ASVs in the bulk fecal matter (average relative abundance > 0.1%) are found in the biobank. Notably, a substantial fraction of the uncultured gut bacteria belonged to the *Ruminococcaceae* and *Lachnospiraceae* families (Fig. 2e and Supplementary Table 6), which has also been previously documented as ‘unculturable’^24^. For each ASV, we compared the number of isolates generated in our total isolate collection versus their average abundance in the bulk feces (Fig. 2f), which appeared to be positively correlated. Still, we identified a set of abundant yet difficult-to-culture bacteria, including *Faecalibacterium* ASV-58, *Prevatella* ASV-470 and ASV-324, *Oscillibacter* ASV-215 and *Clostridium XlVa* ASV-287 (Fig. 2g). Interestingly, *Faecalibacterium* ASV-58, from which we obtained one isolate and performed WGS, matched with >98% genome-wide average nucleotide identity (ANI) to the metagenome-assembled genome (MAG) of *Candidatus cibiobacter qucibialis*. This strain in our collection was previously reported as the most abundant uncultured species in human gut^25^ and is highly depleted in inflammatory bowel disease (IBD) patients, as are other *Faecalibacterium* strains^26^.

We further compared isolates in our biobanks to existing database^1,3,22,25^ by WGS and identified 11 additional species that had not been cultivated in any reference collections (BIO-ML, CGR and HMP) but are only associated with MAGs in the SGB collection (Supplementary Fig. 7 and Supplementary Table 7). For example, besides *Faecalibacterium* ASV-58, we isolated another abundant species *Faecalibacterium sp*. ASV-76 that represents >3% relative abundance on average in the bulk fecal matter, which further expands the collection of culturable gut microbiomes. Together, these results highlight cultured isolates and the remaining missing diversity based on our current media and growth conditions, and offer directions to guide future culturomics efforts focused on these ‘dark matter’ gut microbiome (Supplementary Table 6).

### Taxonomy prediction from morphology enables targeted isolation

**Fig. 3:**
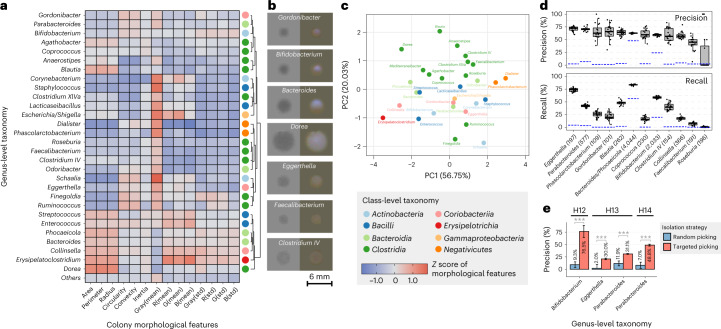
Using colony morphology to predict taxonomic identity enhances targeted isolation. **a**, Heatmap of average *z* scores of morphological features across different bacterial genera. Different genera exhibiting diverse morphological patterns were classified into different groups by hierarchical clustering and the colored dot on the right represents their class-level taxonomy. **b**, Examples of colony images. Transilluminated images are on the left side and epi-illuminated images are on the right side. **c**, PCA ordination of genera based on their colony morphological features. Colors indicate class-level taxonomy. **d**, Performance of bacterial genus prediction based on morphological features by a random forest classifier. The numbers in brackets represent the number of isolates for each genus. Model training and evaluation were bootstrapped 20 times and the box plots show the variance of performance (*n* = 20). Blue line represents the performance of null model. Definition of box-plot elements—center line, median; box limits, upper and lower 25th quartiles; whiskers, 1.5× interquartile range. **e**, Performance of model-based targeted isolation. Bars represent the mean of prediction precision by individual-specific models that were bootstrapped 20 times and error bars represent the standard deviations. *P* values were calculated by two-sided Student’s *t*-test on precisions from *n* = 20 randomly initialized model bootstrapping.

Focused cultivation of bacteria of interest from a microbiome sample can be crucial for mechanistic studies. Unfortunately, we lack the capacity to selectively culture most bacterial species in a specific manner. Consequently, picking a large number of colonies and relying on statistical probability is the only practical solution for obtaining bacteria of interest. This strategy, however, is often too resource-consuming as it may require manually picking thousands of colonies. CAMII offers an ML-guided and automated colony selection method based on linking taxonomical identity to colony morphology and thus could in theory enhance targeted isolation. To test this, we systematically probed our deep gut isolate collection to analyze the relationship between morphologic and genotypic data. Interestingly, colonies of different genera exhibited diverse morphological patterns (Fig. 3a,b). For example, colonies of *Dorea, Bacteroides* and *Collinsella* are generally large and dense but show different circularities (*Collinsella* > *Bacteroides* > *Dorea*), reflecting differences in their growth characteristics. On the other hand, colonies of *Faecalibacterium* are smaller and fainter, in line with our earlier results of their poor culturability. Furthermore, colony morphologies are significantly clustered according to their phylogeny (*P* = 0.008 by PERMANOVA test in Fig. 3c). For instance, most genera of *Clostridia* are closer to each other by morphology-based ordination (Fig. 3c). Therefore, colony morphologies may embed a substantial amount of information that could be linked to taxonomic identities.

We assessed whether taxonomic identity of colonies could be uniquely predicted by only incorporating their morphologic information on plates. We trained a random forest classification model using morphology and taxonomy data from randomly selected subsets of isolates (70% of the total; Methods). The model performance was evaluated on the remaining 30% of isolates. Remarkably, our model achieved ~70% precision for most genera that had more than 100 isolates in the training dataset (Fig. 3d). The recall rate at the genus level varied more widely, highlighting open opportunities to use more sophisticated models to learn additional unique colony features^27–29^. Some genera such as *Eggerthella* had high precision and recall, indicating that highly conserved and unique colony morphologies could be specifically leveraged for taxonomic predictions. When analyzing isolates from the same ASV, we found that colony morphology was highly conserved for isolates within the same person but was much more variable between isolates from different people (Supplementary Fig. 8). Given that different people usually carry distinct strains of the same species, our results suggested a high degree of strain-level variation in colony morphology.

To assess whether AI-informed colony features can improve targeted microbe isolation, we next trained random forest models on our biobank isolates data from three different people separately (H12, H13 and H14). The models were used to predict colonies of *Bifidobacterium*, *Parabacteroides* and *Eggerthella* from new plates derived from the same fecal samples, and the colonies were then isolated by CAMII and 16S rRNA sequenced to confirm taxonomic identity (Methods). Notably, morphology-guided picking substantially improved the isolation efficiency for these targeted genera by up to eightfold on average (Fig. 3e), largely increasing the precision of picking and mitigating the need to screen many colonies to find the desired microbes. These results emphasize the value of our biobank datasets that link phenotype to genotype and demonstrate taxonomic predictions from visual colony features alone, which can greatly enhance targeted microbial isolation.

### Interbacterial growth associations between gut microbiota

**Fig. 4:**
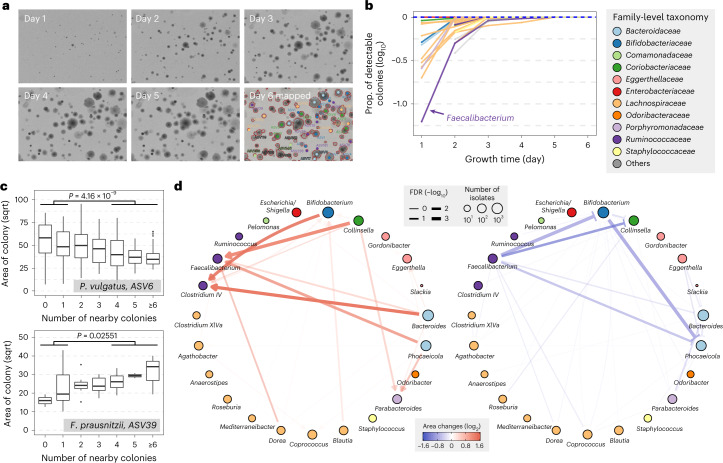
Mapping interaction between gut microbiota by colony morphology analysis. **a**, Images of an example plate during 6 d of growth and colony identities on the plate by 16S sequencing. **b**, Proportion of detectable colonies at different time points compared to day 6 for each genus. Colors indicate the family-level taxonomy. **c**, Correlation of colony size and number of nearby colonies for two representative ASVs. A full list of correlations is provided in Supplementary Table 8. *P* values are calculated by one-sided Mann–Whitney *U* test on area of colonies with no more than one nearby colony or no less than four nearby colonies (*n* = 101 versus 82 for ASV-6 and 17 versus 9 for ASV-39). Definition of box-plot elements: center line: median; box limits: upper and lower 25th quartiles; whiskers: 1.5× interquartile range. **d**, Pairwise growth promoting and inhibiting networks between genera. Directional growth-promoting effects are shown in red sharp arrows and directional growth-inhibiting effects are shown in blue blunt arrows. Nodes represent bacterial genera and are colored by family. Node sizes are proportional to the number of isolates used in this analysis and edges widths are proportional to the significance of the interactions.

Bacterial colonies can influence the growth of their neighbors through species interactions such as competing for nutrients or cross-feeding essential metabolites. Previous studies suggest that neighboring cells can critically affect the size of colonies in a predictable manner^19^. Because CAMII can track the kinetic growth of colonies continuously, we systematically probed the cogrowth associations between gut isolates on agar plates. A fecal sample (H1t5; Supplementary Table 4) was plated and imaged daily, and all colonies were subsequently isolated on day 6 and their taxonomic identities were determined with 16S sequencing (Fig. 4a). For each ASV, the cumulative area of colonies on agar plates correlated with their abundances in the original fecal sample (Supplementary Fig. 9), indicating that our in vitro conditions generally fostered growth to the same degree as in the gut. Interestingly, colonies belonging to the *Faecalibacterium* genus exhibited slower initial growth and only began to emerge in the presence of other nearby growing colonies (Fig. 4b; Methods). This observation suggests that commensal or mutualistic interactions may be at play between *Faecalibacterium* and other species.

To more systematically study species interactions enabled by CAMII, we analyzed the colony morphology, taxonomic identity and colony neighborhood data together. We aggregated morphology data and physical coordinates from 102,071 visually captured colonies (26,997 isolated) and assessed whether a colony’s growth is affected by neighboring cells. Surprisingly, we observed a number of interesting cogrowth patterns that may reflect interspecies interactions (Supplementary Table 8). For example, the colony size of *Phocaeicola vulgatus* ASV-6 is negatively correlated with the number of neighboring cells, consistent with a scenario that there are general negative interactions mediated by competition or antagonism between *P. vulgatus* and other bacteria in the gut^30^ (Fig. 4c). On the other hand, *Faecalibacterium prausnitzii* ASV-39, one of the species associated with slower initial growth in colony kinetics (Fig. 4b), grew larger colonies with more neighbors reflective of a positive species interaction (Fig. 4c).

We next incorporated taxonomic information of nearby colonies and looked at how the colony size of a specific genus could be affected by other genera. Briefly, for each pair of genera, we compared the colony sizes of one genus with the other genus present in the neighborhood and without any colonies present (Methods). Remarkably, we identified isolates from two genera, *Faecalibacterium* and *Clostridium IV*, that grow into larger sizes when the isolates were close to *Bifidobacterium*, *Phocaeicola* and *Bacteroides* (Fig. 4d). *Faecalibacterium* and *Clostridium IV* have been reported to be major butyrate-producing bacteria in the gut and could benefit from coculture growth with *Bifidobacterium* and *Bacteroides* species^31–33^, which is consistent with our findings. On the other hand, we observed that *Phocaeicola* isolates are smaller with *Faecalibacterium* isolates as neighbors (Fig. 4d), indicating that the cogrowth interaction might be beneficial to only one side. Furthermore, consistent with our previous correlation analysis that examined neighboring isolate numbers without the consideration of neighbors’ identity, we observed that the growth of *Phocaeicola* and *Bacteroides* could be inhibited by multiple other genera, suggesting further investigations to better understand the underlying mechanism of these positive and negative interactions between gut microbiota. Together, our results highlight that CAMII can reveal colony cogrowth patterns governed by interspecies interactions, which may help identify growth-promoting microbes and their diffusible metabolites that stimulate in vitro growth of fastidious species.

### Intra- and interpersonal genomic diversity of gut strains

Mapping the strain-level genome-wide diversity of gut bacteria within a person is important for understanding the dynamics of gut colonization and the drivers of bacterial selection and adaptation specific to each human host^1,2,34^. A key advantage of the CAMII system is the ability to isolate and perform WGS for a large number of isolates to help investigate inter- and intrapersonal genomic variations. As such, we selected isolates covering the most unique and prevalent ASVs from our 20-person microbiome biobank and performed WGS that yielded 1,197 high-quality draft genomes (Supplementary Fig. 10 and Supplementary Table 9). Genome assemblies were further analyzed to determine the accurate species-level taxonomy of isolates (Methods).

**Fig. 5:**
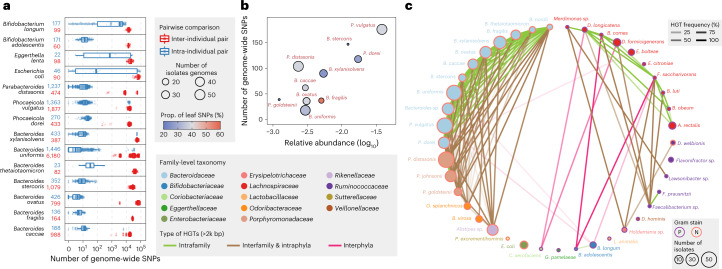
Strain-level genomic diversity of the gut microbiome within and between individuals. **a**, Number of genome-wide SNPs between isolates from the same or different individuals for 14 isolates-rich species. The numbers after the species name represent the number of inter-individual (red) and intra-individual (blue) pairs. Definition of box-plot elements: center line: median; box limits: upper and lower 25th quartiles; whiskers: 1.5× interquartile range. **b**, Correlation between number of genome-wide SNPs and relative abundance in original fecal sample for isolates-rich species in individual H1. The size of dot represents number of isolates used in this analysis and the color represents proportion of SNPs present in only one genotype. **c**, Network of 2 kb + HGT frequency based on 409 isolates from H1. Nodes represent bacterial species and are colored by family. Node sizes are proportional to the number of isolates in the H1 collection and edges opacity are proportional to the HGT frequency between the two connected species. Edge color represents different types of HGT, that is, interphyla, intraphyla and interfamily, or intrafamily HGT and node outline color represent Gram staining of the species.

We first explored the interpersonal strain-level genomic variations across our isolate collection (Methods). Consistent with previous reports^1,35^, most isolates within the same individuals had very few genomic variations (that is, less than 10^2^ SNPs) while isolates between people differed by 10^3^–10^5^ genome-wide SNPs (Fig. 5a). Interestingly, some phylogenetically distinct isolates (that is, more than 10^4^ SNPs) of the same species were observed to coexist within the same person (Fig. 5a). For instance, two distinct strains of *P. vulgatus* were isolated from the H4 individual and two distinct strains of *B. uniformis* were found in the H2 individual (Supplementary Fig. 11).

We next sought to assess the strain-level diversity within a single person by analyzing 408 isolate genomes derived from the H1 individual (Supplementary Fig. 10; Methods). Because abundant species in the gut are expected to undergo more cell divisions, we hypothesized that they may accumulate more SNPs across their genomes, assuming approximately the same duration of gut colonization. Indeed, the number of genome-wide SNPs within each taxon is generally correlated with its abundance in the original microbiome (Fig. 5b). *B. fragilis* shows a higher proportion (56.0%) of leaf SNPs (that is, present in only one genotype) while other species show much lower proportions, including *P. goldsteinii* (20.5%), *B. stercoris* (22.4%) and *B. xylanisolvens* (25.6%), which suggests differential population bottlenecks and selective sweeps at the species level. At the gene level, we also observed evidence of convergent adaptive evolution. For instance, between different *P. dorei* isolate lineages, we identified two coding variants in gene *TodS* (Supplementary Fig. 12), which encodes a two-component kinase sensor regulating toluene metabolism in bacteria^36^. Toluene and other aromatic hydrocarbons are found in foods and are also used as industrial feedstocks that could contaminate foods and thus drive evolution in the gut^37^.

Another major driver of within-person gut microbiome evolution is HGT. Accordingly, we used all whole-genome sequenced H1 isolates to reconstruct an HGT network of shared DNA elements >2 kb in length (Methods). Consistent with recent reports^38,39^, we observed that HGT events were strongly linked to the phylogeny of the isolates, that is, most HGT events occurred within the same phyla but were also quite prevalent across different families and between distinct species (Fig. 5c and Supplementary Table 10). Interestingly, we observed that HGTs were predominantly enriched between isolates with the same Gram staining, with Gram-negative species showing more prevalent HGTs than Gram-positive species (*P* = 0.0005 by Pearson’s chi-squared test). This result is consistent with recent finding^39^ and suggests that different cell wall structures may play an important role in HGTs. Notably, HGTs between Gram-positive and -negative species were also observed in our dataset, inspiring future studies to study the effect of cell wall structures on HGTs and engineer these HGT elements into a microbiome editing tool. Next, to examine whether these HGTs occurred recently, we calculated the mean HGT frequency between all species pairs (Methods). We hypothesized that if HGTs occurred recently between two species, they would be only associated with a small proportion of isolates, resulting in a low frequency between species, while if HGTs occurred earlier and provided growth benefits, they would be enriched and vertically inherited by later generation, resulting in a high frequency. Interestingly, we found most HGT elements were frequently present across isolates (71.5% HGTs with >50% frequency), especially for ones within *Bacteroidaceae* species (Fig. 5c), suggesting that they occurred in the distant past and were enriched under strong selection within the gut environment.

Given the high prevalence and frequency of within-individual HGT, we next annotated the protein-coding sequences of the most widespread HGT elements to probe their potential functions (Methods). Interestingly, we identified multiple antibiotic resistance genes (ARGs) with different mechanisms of action as well as secretion system genes (Supplementary Fig. 13). For example, the top four most widespread HGT sequences are found surprisingly in at least 13 different species of *Bacteroidaceae*, *Porphyromonadaceae*, *Odoribacteraceae,* and *Rikenellaceae* and contained multiple ARGs including ribosomal protectors and antibiotic efflux pumps, as well as type III and type IV secretion systems. While ARGs and secretion systems shared through HGT may confer clear evolutionary advantages^40,41^, there were numerous widespread elements across different species with genes of unknown function (Supplementary Fig. 13), hinting at unexplored mechanisms that drive their long-term persistence in the gut. Taken together, these results highlight that isolates within and across people have genomic diversity that can be systematically characterized using CAMII-enabled deep strain biobanking and genomic analysis to study person-specific gut microbiome colonization, adaptation and ecology.

## Discussion

Strain isolation from the gut microbiome has historically been performed in an ad hoc manner where important phenotypic features are inadequately captured and poorly documented alongside genomic data. Here we described the CAMII platform to industrialize the generation of isolate biobanks by leveraging automation, machine vision, supervised learning and genomics. When combined with low-cost 16S and whole-genome sequencing, the systematically generated phenotypic and genomic data produced from the pipeline forms a rich resource to study microbial colony morphology, diversity and evolution. Using the gut microbiome as a showcase example, CAMII-enabled isolation yielded extensive isolate biobanks from 20 healthy individuals that in aggregate covered >80% of all microbiota by abundance present. This isolate collection covers a majority of microbial diversity in the healthy gut and is one of the most extensive personalized isolate biobanks described to date. Using this resource, we demonstrated that quantitative analysis of colony morphologies can predict taxonomy, enhance the isolation of targeted genera and reveal potential interactions between microbes. Systematic analysis of genomic differences between isolates within and across people revealed interesting patterns of population selection, adaptation and HGT.

The majority of the data presented here relied on a common mGAM-rich media for strain isolation and characterization in the context of the human gut microbiome. Exploration of alternative media formulations, other micronutrients and macronutrients, and host or environmentally associated biochemical perturbations (for example, bile acids and xenobiotic compounds) could yield morphologic and growth profile changes that inform unexplored physiologies and characteristics of the gut microbiome. The interspecies interactions derived from CAMII datasets could be further used to systematically map out the drivers of microbiome dynamics. We envision that these interactions could facilitate cultivation of recalcitrant ‘dark matter’ microbiome by helping to identify unknown microbially-derived molecules that promote cooperative growth observed in this and other studies.

The CAMII system uses commercially available off-the-shelf components and open-source code that can be readily replicated by other researchers (see Supplementary Table 1 for list of components). We envision that the searchable online portal will facilitate sharing of standardized phenotypic and genomic data, which is poised to grow over time. The CAMII hardware could be further expanded to integrate mass spectrometry measurements to gain additional colony characteristic profiles that can improve species and metabolite identification. Onboard automated microscopy could further introduce orthogonal data streams to visualize microbial cells at micrometer resolution across different spectral channels. Improved machine vision and ML algorithms could yield even better strain predictions and enhance isolation performance.

Because individual strains are the unit of action within a complex community, more complete strain collections are needed. Such comprehensive biobanks can be used to recreate a more holistic context that takes into account the composition, interspecies interactions and metabolic capacity of the entire community, which will improve studies of microbiome function, dynamics and stability. Beyond the human gut, CAMII can be useful for other microbiomes such as those from soil, aquatic or agricultural settings, including further isolation and analysis of phages, fungi and protozoa. The robotic automation system can also help generate systematic strain libraries such as arrayed transposon insertion knock-out collections^42^ or functional genomics expression libraries^43^ as well as improve screening for tractable microbial chassis for genetic engineering^44^.

## Methods

### Ethical review

This study was approved and conducted under Columbia University Medical Center Institutional Review Board protocol AAAR0753. Written informed consent was obtained from participants in the study.

### Fecal sample collection and storage

Fresh fecal samples were collected from 20 healthy human donors and processed within 3 h of defecation. Briefly, feces were collected using the Commode Specimen Collection System (Fisher, 02-544-208). An inverted sterile 200-µl pipette tip (Rainin, RT-L200F) was used to core out a small sample from the stool specimen, which was then immediately placed in a sterile cryovial (Fisher, NC9347001). The collected fecal samples were then transferred to an anaerobic chamber (Coy Laboratory) and homogenized in 5 ml of prereduced PBS by thorough vortexing. Homogenized samples were further passed through a 40-µ filter (Fisher, 22363547) to remove dietary debris, aliquoted into multiple cryovials with glycerol (20% final concentration) and transferred to a −80 °C freezer for long-term storage.

### Plate preparation and bacterial culture

All gut microbiota were grown in Gifu Anaerobic Medium Broth, Modified (mGAM; HyServe, 05433) under anaerobic conditions (5% H_2_, 10% CO_2_ and 85% N_2_) in an anaerobic chamber. Briefly, 1.5% agar plates (Thermo Fisher Scientific, 242811) with mGAM media were made using a peristaltic pump (New Era Pump Systems NE-9000) and labeled with unique barcodes. For plates supplemented with ciprofloxacin (10 µg ml^−1^), trimethoprim (50 µg ml^−1^) or vancomycin (50 µg ml^−1^), antibiotics were added during plate preparation. All plates were then transferred to the anaerobic chamber and prereduced for ~24 h before plating. Frozen fecal samples were thawed in the anaerobic chamber and diluted to 10^3^ CFU per ml for each culturing condition. Optimal dilutions were determined by sample-specific serial dilution experiments. Two hundred microliters of diluted fecal samples was then dispensed onto the plate and spread using sterile glass beads. Plates were sealed in Ziploc bags to reduce desiccation and incubated at 37 °C for 5 d of colony growth.

### Strain imaging and isolation

Strain imaging and isolation was performed using a custom automated imaging and colony-picking system (CAMII). After 5 d of growth, agar plates were imaged automatically on the CAMII system (Fig. 1c). Briefly, plates were first placed on a carrousel stacker. A robotic arm gripper carried individual plates past a barcode scanner to an illuminated imaging platform on the colony picker where they were imaged under two lighting conditions (epi-illumination and transillumination) by the Hudson RapidPick control software. The plate labels are linked to the captured images and imaged plates were automatically restacked by the robotic arm. Following completion of the imaging process, plates were sealed in Ziploc bags to avoid desiccation and a custom script was used to segment different colonies and identify morphologically unique colonies for subsequent picking based on plate images (Supplementary Fig. 1a). Morphologic features include area, perimeter, mean radius, circularity, convexity, inertia and mean and variances along gray channel (transilluminated images) and RGB channels (epi-illuminated images). Raw images of all colonies on the plate are also collected.

For random picking performed in this study, a random subset of a given number of colonies was generated from all detected colonies by the script and the automatic isolation was performed on these colonies. For phenotype-guided picking, all detected colonies were first subjected to optimized selection based on their morphology, and a subset of a given number of colonies with maximized morphological diversity was isolated by CAMII. Detailed algorithm of optimized colony selection can be found in Supplementary Fig. 1b, and scripts used to analyze plate images and colony morphologies can be accessed at https://github.com/hym0405/CAMII.

After analyzing plate images and generating a list of colonies to pick, a similar robotic protocol was executed to isolate these colonies. Firstly, plates were restacked for picking and a multichannel media dispenser was used to dispense 50 µl of mGAM liquid media into each well of two barcoded sterile 384-well optical plates (Thermo Fisher Scientific, 12-566-2; duplicate ‘A’ and ‘B’), which were then moved to the colony picker. Next, an agar plate was transferred to the colony picker and heat-sterilized needles picked individual colonies into the duplicate optical plates. Plates were automatically switched out when all targeted colonies were picked (agar plate) or all wells were inoculated (optical plate). After colony picking, inoculated optical plates were transferred to a plate sealer (Brandel, 9795), sealed and restacked. Optical plates were then incubated at 37 °C for ~5 d for bacteria culturing. After bacterial growth, ‘A’ plates were subjected to downstream gDNA extraction and 30 µl of 40% glycerol was added to each well of ‘B’ plates, which were transferred to −80 °C for long-term storage.

### Colony morphology analysis

To achieve morphology-guide colony selection, colony morphological features extracted in raw image processing were centralized and scaled to unit variance and then embedded by PCA. An optimized colony selection algorithm was further applied to embedded features to search a set of colonies with most morphological diversity (Supplementary Fig. 1b).

To evaluate how different ASVs respond to nearby colonies (Fig. 4c), number of nearby colonies were calculated for isolates on plates, and ‘nearby colony’ pair was defined as two colonies with distance between their *X*–*Y* coordinates shorter than 30 pixels plus the sum of their radii. To avoid potential impact of antibiotics on colony morphology, only colonies grown on mGAM-only plates were used for morphology analysis.

To evaluate how the growth of a specific genus could be affected by other genera (Fig. 4d), we first identified ‘nearby colony’ pairs as described above, and the growth impact of genus-A on genus-B is quantified by comparing the colony sizes of genus-B with genus-A present in the neighborhood, that is, as nearby colony, to the colony sizes of genus-B without any nearby colonies: the effect size was defined as the fold-change of average colony size with genus-A present to average colony size without any nearby colonies, and the *P* values were calculated by Mann–Whitney *U* test on the size distributions between with genus-A present in the neighborhood and without any nearby colonies, and false discovery rate (FDR) correction was performed using Bonferroni–Holm methods. To avoid potential impact of antibiotics on colony morphology, only colonies grown on mGAM-only plates were used for morphology analysis.

### Taxonomy prediction and targeted isolation

To test whether colony morphology on plates could help predict taxonomic identity, colonies of data-rich genus (>100 isolates across all 20 individuals) were subjected to model training and testing. Considering the potential impact of antibiotics perturbation and neighbor colonies, a multilabel random forest model was trained on 70% of isolates, which was randomly sampled using 14 colony morphological features, antibiotics condition and number of nearby colonies, and the performance (precision and recall) of the model was evaluated on the remaining 30% of isolates. The procedure of model training and evaluating was bootstrapped 20 times with different randomization settings to minimize bias, and the background performance of the model was calculated by null model (prediction based on the number of isolates). To perform targeted microbial isolation, a multilabel random forest model was trained on colonies of data-rich genus (>15 isolates from the same individual) from individuals H12, H13 and H14 separately as described above. The same fecal samples were then plated out and the model was applied to the new plates after bacterial growth to screen all colonies on plates and predict colonies of targeted genus-level taxonomy. All colonies of the plates were then isolated on CAMII and subjected to 16S V4 sequencing to identify their taxonomy and evaluate the performance of targeted isolation.

### Daily kinetic growth analysis of colonies on plate

To monitor the growth kinetics on a daily basis, fecal sample H1t5 was plated out on mGAM-only plates and the plates were imaged every day during 6 d of growth. Colony detection and segmentation were performed on images, and colony morphology features on different days were matched based on their *X*–*Y* coordinates (Fig. 4a). All colonies on plates were then isolated on CAMII on day 6 and subjected to taxonomy identification by 16S rRNA sequencing. To quantify differential initial growth of genera, the number of detectable colonies of each genus on each day (tracked by *x*–*y* coordinates) was normalized to their total number of colonies on day 6 to calculate the proportion of detectable colonies (Fig. 4b) at each day.

### gDNA extraction

gDNA of picked isolates were extracted in 384-well format using a silica bead beating-based protocol adapted from a prior study^45^. Firstly, 40 µl 0.1 mm Zirconia Silica beads (Biospec, 11079101Z) and 120 µl lysis solution (50 mM Tris–HCl, pH 7.5 and 0.2 mM EDTA) were added to each well of 384-well deep-well plates (Thermo Fisher Scientific, 07-202-505). Next, 40 µl culture solutions of isolates were added to each well and the plates were centrifuged for 1 min at 4,500*g* and affixed with a sealing mat (Axygen, AM-384-DW-SQ). To avoid overheating during bead beating, the plates were vortexed for 5 s and incubated at −20 °C for 10 min before beating. Then, plates were fixed on a bead beater (Biospec, 1001) and subjected to bead beating for 5 min, followed by a 10-min cooling period. The bead beating cycle was repeated once and plates were centrifuged at 4,500*g* for 5 min to spin down cell debris. Next, 10 µl cell lysate was transferred to a 384-well PCR plate (Bio-Rad, HSP3801) and 2 µl proteinase K solution (50 mM Tris–HCl, pH 7.5 and 1 µg µl^−1^ proteinase K (Lucigen, MPRK092)) was added using a Formulatrix Mantis. Finally, cell lysate was subjected to proteinase K digestion on a thermal cycler (65 °C 30 min, 95 °C 30 min, 4 °C infinite) and transferred to −20 °C for long-term storage. gDNA extraction for bulk feces samples was performed using the same protocol with scale-up reaction volumes in 96-well format.

### 16S rRNA amplicon sequencing

16S sequencing of the V4 region for isolates taxonomy identification was performed in 384-well format using a set of dual-indexing sequencing primers. Briefly, barcoded 16S V4 amplicon primers were designed based on universal 16S V4 primers and synthesized by Integrated DNA Technologies. Next, 1 µl of each unique combination of barcoded forward primer 16SV4f_5xx and reverse primer 16SV4r_7xx were transferred to a 384-well PCR plate using Labcyte Echo to make unique dual-indexed primer plates. Then, ~130 nl of gDNA was transferred to a primer plate by a 384-well pin replicator (Scinomix, SCI-6010.OS) and 2 µl NEBNext Q5 PCR master mix (NEB, M0543L) was added to each well using Formulatrix Mantis. The samples were then subjected to 16S V4 amplification on a thermal cycler (98 °C 30 s, 40 cycles: 98 °C 10 s, 55 °C 20 s, 65 °C 60 s; 65 °C 5 min; 4 °C infinite). The resulting amplicon libraries were manually pooled and subjected to gel electrophoresis on E-Gel EX Agarose Gels, 2% (Thermo Fisher Scientific, G402002). Expected DNA bands (~390 bp) were excised from gel and extracted by Wizard SV Gel and PCR Cleanup System (Promega, A9282) following the manufacturer’s instructions to remove PCR primers and adapter dimers. Gel-purified libraries were quantified by Qubit dsDNA HS assay (Thermo Fisher Scientific, Q32851) and sequenced on Illumina MiSeq platform (reagent kits: v2 300-cycles, paired-end mode) at 8 pM loading concentration with 20% PhiX spike-in (Illumina, FC-110-3001) along with custom sequencing primers spiked into Miseq reagent cartridge (6 µl of 100 µM stock; well 12: 16SV4_read1, well 13: 16SV4_index1, well 14: 16SV4_read2) following the manufacturer’s instructions. Sequences of all primers used in library preparation and sequencing are provided in Supplementary Table 11. 16S V4 sequencing of the bulk samples was performed using similar protocol with scale-up reaction volumes in 96-well format. Moreover, SYBR Green I (final concentration: 0.2×; Thermo Fisher Scientific, S7563) was added to the PCR reaction and a quantitative 16S V4 amplification was performed and stopped during the exponential phase (typically 13–17 cycles) and the reaction was advanced to the final extension step.

### 16S rRNA amplicon analysis and ASV clustering

Raw sequencing reads of 16S V4 amplicon were analyzed by USEARCH v11.0.667 (ref. ^46^). Specifically, paired-end reads were merged using ‘-fastq_mergepairs’ mode with the default setting. Merged reads were then subjected to quality filtering using ‘-fastq_filter’ mode with the option ‘-fastq_maxee 1.0 -fastq_minlen 240’ to only keep reads with less than one expected error base and greater than 240 bp. Remaining reads were deduplicated (-fastx_uniques) and clustered into ASVs (-unoise3) at 100% identity, and merged reads were then searched against ASV sequences (-otutab) to generate ASV count table. Taxonomy of ASVs was assigned using Ribosomal Database Project classifier v2.13 trained with 16S rRNA training set 18 (ref. ^47^). Relative abundance of ASVs in bulk samples is defined as reads count of ASVs normalized by total number of mapped reads.

### Isolate taxonomy identification and 16S phylogeny analysis

After ASV clustering, ASV count table was parsed to calculate the following metrics for each isolate: total reads count, the ASVs with the highest reads count and purity of that ASV. Isolates with insufficient reads or poor purity (reads counts < 5 or purity < 0.5) were filtered and the taxonomy of remaining isolates were defined as the ASVs with the highest reads count. To construct the phylogeny of isolates, multisequence alignment was performed on ASV sequences of the isolates using MUSCLE v5 (ref. ^48^) and aligned ASV sequences were subsequently analyzed by MEGA v11.0.11 (ref. ^49^) to calculate neighbor-joining tree with the default setting for phylogeny reconstruction.

### Isolates whole-genome sequencing and reads processing

The same gDNA used for 16S V4 amplicon sequencing was subjected to whole-genome sequencing for isolates. Paired-end libraries were constructed following a published protocol of low-volume Nextera library preparation^50^ and sequenced on Illumina Nextseq 500/550 platform (2 × 75 bp) and HiSeq platform (2 × 150 bp). Raw reads were then processed by Cutadapt v2.1 with the following parameters: ‘--minimum-length 25:25 -u 10 -u -5 -U 10 -U -5 -q 15 --max-n 0 --pair-filter=any’ to remove low-quality bases and Nextera adapters. Coverage was 1.42 ± 2.86 million paired-end reads per isolate and PacBio long-read sequencing was performed for some isolates by SNPsaurus to improve the performance of de novo genome assembling.

### De novo genome assembling and SNP variation analysis

Illumina reads passing quality filtering and PacBio long reads were assembled by Unicycler v0.4.4 (ref. ^51^) with default setting to generate draft genomes of isolates, and the quality and species-level taxonomy of draft genomes were then assessed by QUAST v4.6.3 (ref. ^52^), CheckM v1.0.13 (ref. ^53^) and GTDB-Tk v0.2.2 (ref. ^54^). Among all 3,271 isolates assemblies, 1,197 were defined as high-quality draft genomes (coverage > 20×; N50 > 5,000 bp; completeness > 80%; contamination < 5%) and used for downstream genomic variation and HGT analysis. To identify strain-level genomic variation of gut microbiota isolates within and between individuals, draft assemblies with the highest completeness and N50 of each species were selected as the reference genomes for reads alignment, and processed Illumina reads of isolates were aligned to reference genomes of the same species by Bowtie2 v2.3.4 (ref. ^55^) in paired-end mode with ‘--very-sensitive’ setting. Resulting reads alignments were then processed by SAMtools v1.9 and BCFtools v1.9 (ref. ^56^) with ‘--ploidy 1’ setting to call genomic variation (SNPs and Indels). Resulting variations were then subjected to quality filtering to identify ‘reliable’ genotypes (covered by ≥5 reads; with ≥0.9 haploidy) and only SNP variations with more than 90% ‘reliable’ genotypes across all isolates were used for downstream analysis. To construct SNP-based phylogeny, base profiles of isolates at SNP sites were concatenated together and UPGMA tree was then calculated by MEGA v11.0.11 with the default setting.

### Genome-wide ANI calculation

To identify species isolated in our biobank that had not been cultivated previously, the average nucleotide identity between draft genomes obtained in this study and MAGs or isolates genomes from publicly available databases were calculated by FastANI v1.0 (ref. ^57^), and genomes with >95% ANI were considered to be the same species.

### HGT identification and annotation

To identity HGT occurring between species within H1 isolates, we compared all genomes pairs of different species by BLASTN v2.7.1 (ref. ^58^) with ‘-evalue 0.1 -perc_identity 99’ setting to systematically screen blocks of genomic regions with high sequence identity. The *P* value of candidate HGTs was then calculated based on the genome-wide ANI between isolates and further adjusted by Benjamini–Hochberg procedure. Blast hits with adjusted *P* value <1 × 10^−5^ and larger than 2,000 bp in length were considered as HGT events between isolates of different species. The frequency of HGTs between species was quantified using a previously published method^39,59^, defined as the number of between-species genome pairs that share at least one HGT divided by the total number of between-species genome pairs. To annotate ARGs and secretion systems in HGT elements, sequences of HGT elements were annotated by Prokka v1.12 (ref. ^60^) in metagenome mode and resulting CDS were searched against CARD database v3.1.4 (ref. ^61^) by BLASTP v2.7.1 to identify ARG hits with *e* value <1 × 10^−5^, identity >20 and query coverage >50. Secretion systems were also predicted on CDS of HGT elements by EffectiveDB^62^ with the default setting.

### Reporting summary

Further information on research design is available in the Nature Portfolio Reporting Summary linked to this article.

## Online content

Any methods, additional references, Nature Portfolio reporting summaries, source data, extended data, supplementary information, acknowledgements, peer review information; details of author contributions and competing interests; and statements of data and code availability are available at 10.1038/s41587-023-01674-2.

### Supplementary information

Supplementary Information Supplementary Figs. 1–13.

Reporting Summary

Supplementary Tables Supplementary Tables 1–11.

## Data Availability

The sequencing data generated in this study have been submitted to the NCBI BioProject database (http://www.ncbi.nlm.nih.gov/bioproject/) under accession number PRJNA745993 (ref. ^63^). Other associated data of the isolate collection, including morphological features and raw images, can be accessed at http://microbial-culturomics.com. Taxonomy of ASVs was assigned based on 16S rRNA training set 18 provided by Ribosomal Database Project. The annotation of ARG genes and secretion systems in HGT elements was based on CARD database v3.1.4 and EffectiveDB database, respectively.

## Code availability

Scripts used to analyze plate images in this study can be accessed at https://github.com/hym0405/CAMII ref. ^64^.
