# Supplementary material for: High-throughput microbial culturomics using automation and machine learning
Source: Nat Biotechnol. 2023 Feb 20;41(10):1424–33. doi: 10.1038/s41587-023-01674-2 (PMC10567565; doi:10.1038/s41587-023-01674-2)
Supplement: Supplementary file 1 — Supplementary Figs. 1–13. [file 41587_2023_1674_MOESM1_ESM.pdf]

nature biotechnology

Article

https://doi.org/10.1038/s41587-023-01674-2

High-throughput microbial culturomics using automation and machine learning

In the format provided by the authors and unedited

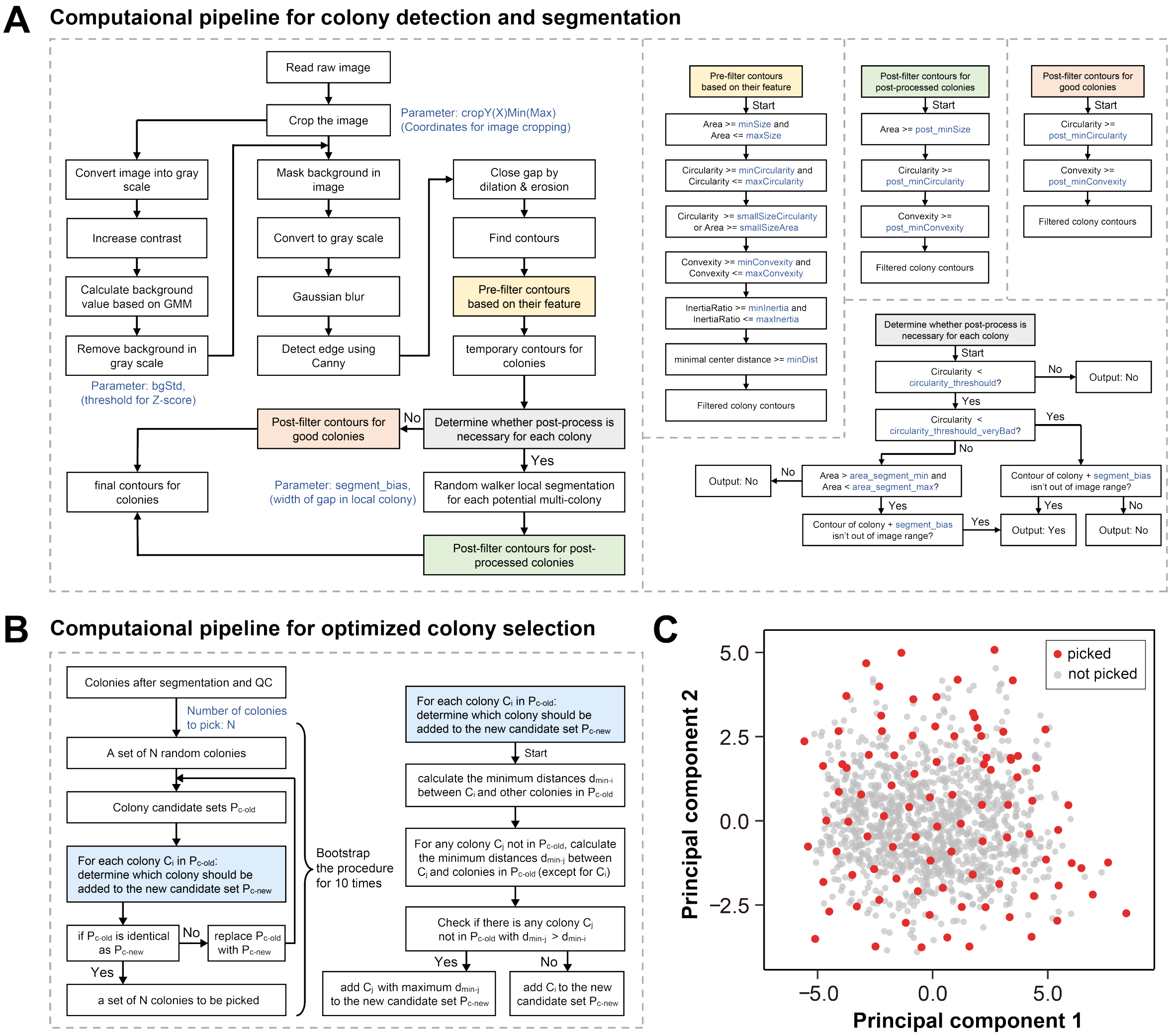

Figure S1: Computational pipeline for image processing and morphology-guide colony selection.

(A) Computational pipeline for colony detection, segmentation, and features extraction on raw images.

(B) Computational pipeline for morphology-guide optimized colony selection on plate.

(C) Colonies are embedded in two-dimension space by PCA based on their morphological features. Dots in red show the colonies selected by the algorithm that maximize the diversity of colony morphology.

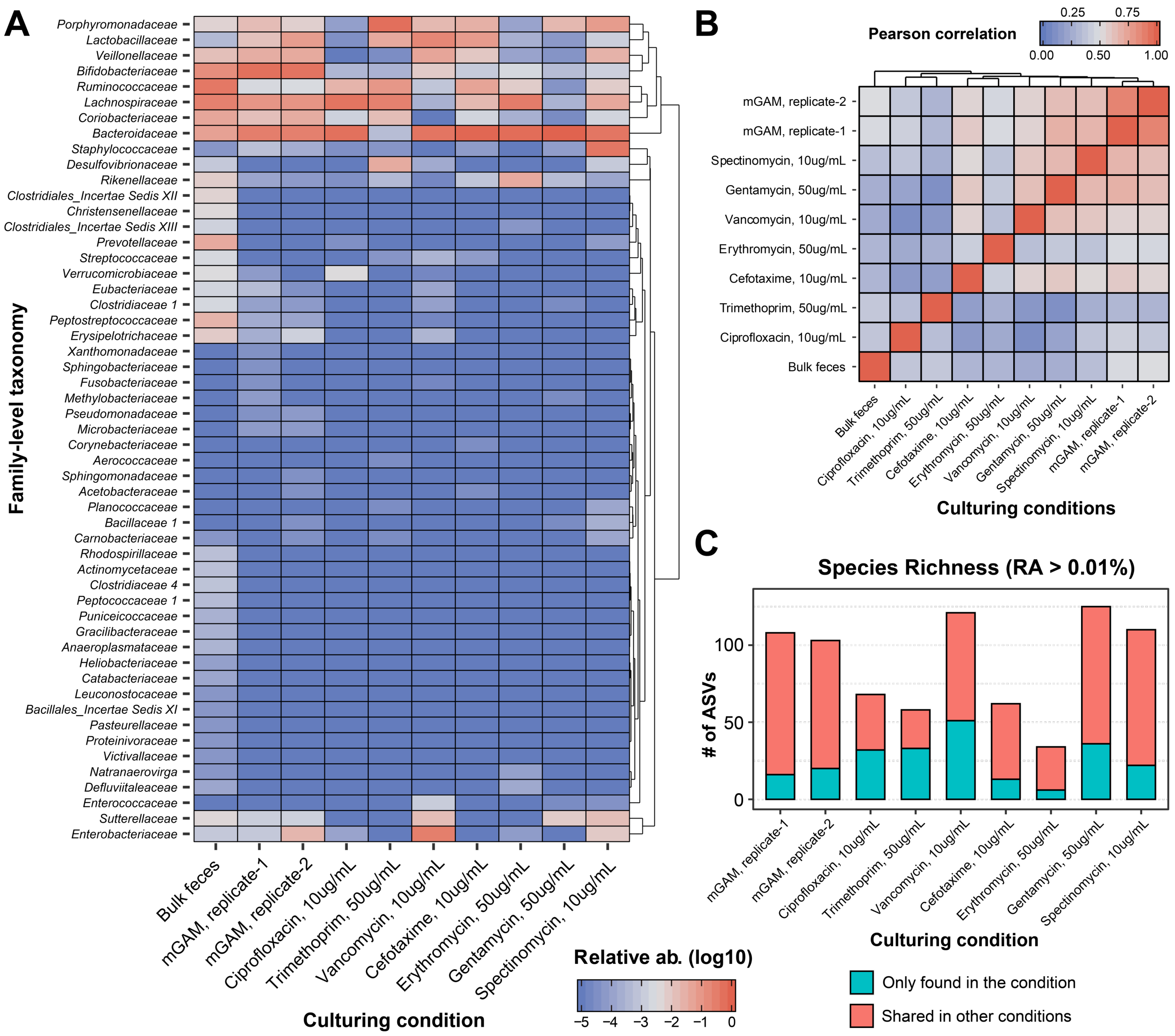

Figure S2: Evaluation of different antibiotics to enrich most unique and diverse microbes.

(A) Heatmap of family-level relative abundance for input feces sample and plate scrape of cultures under different conditions.

(B) Pearson correlation of different culturing conditions based on their yielding ASV-level abundance profiles.

(C) Number of unique ASVs yielded by different culturing condition. ASVs with relative abundance > 0.01% are shown.

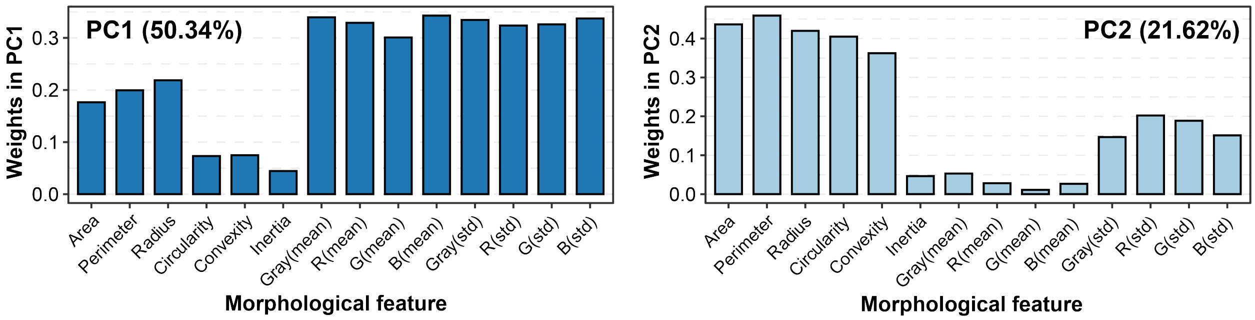

Figure S3: Informative features in PCA analysis of colony morphology.

Weights of morphological features for principal component-1 (left panel) and principal component-2 (right panel) that counts for 50.34% and 21.62% of total variance in features, respectively.

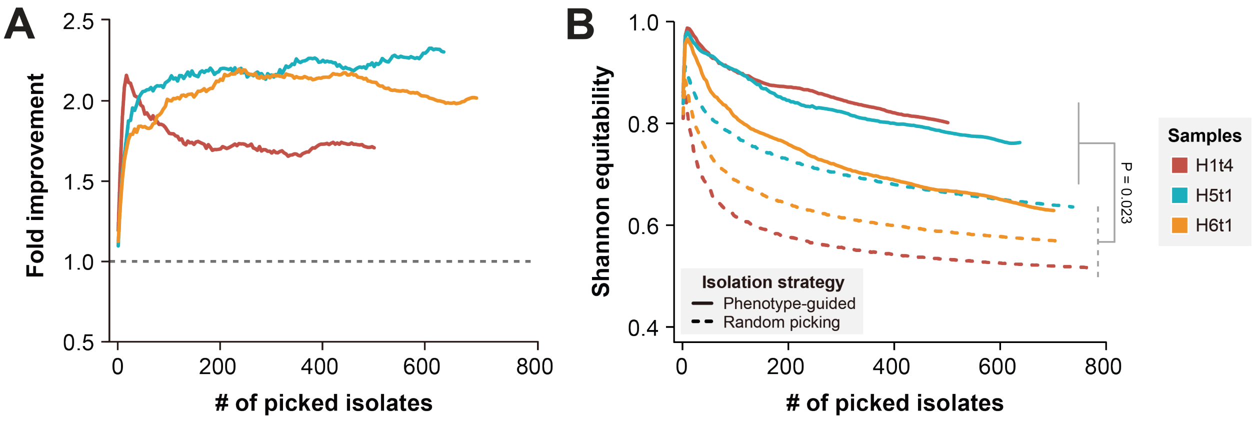

Figure S4. Phenotype-guided isolation robustly yielded more diverse isolates.

(A, B) Performance of phenotype-guided isolation compared to random isolation for three human fecal samples H1t4, H5t1 and H6t1. Fold improvement of unique ASVs counts **(A)** and Shannon equitability of isolates **(B)** were calculated for different number of picked isolates and phenotype-guided strategy exhibited substantial and sustainable improvement in isolating more diverse and unique ASVs. Horizontal line in fold improvement **(A)** represents no increase in number of unique ASVs. Isolation was performed by CAMII automation; Random isolation was performed on a random subset of all detected colonies on the plates and phenotype-guided isolation was performed on morphology-selected colonies by the algorithm (**Figure S1B**). P-value was calculated by two-sided paired t-test on area under the curve.

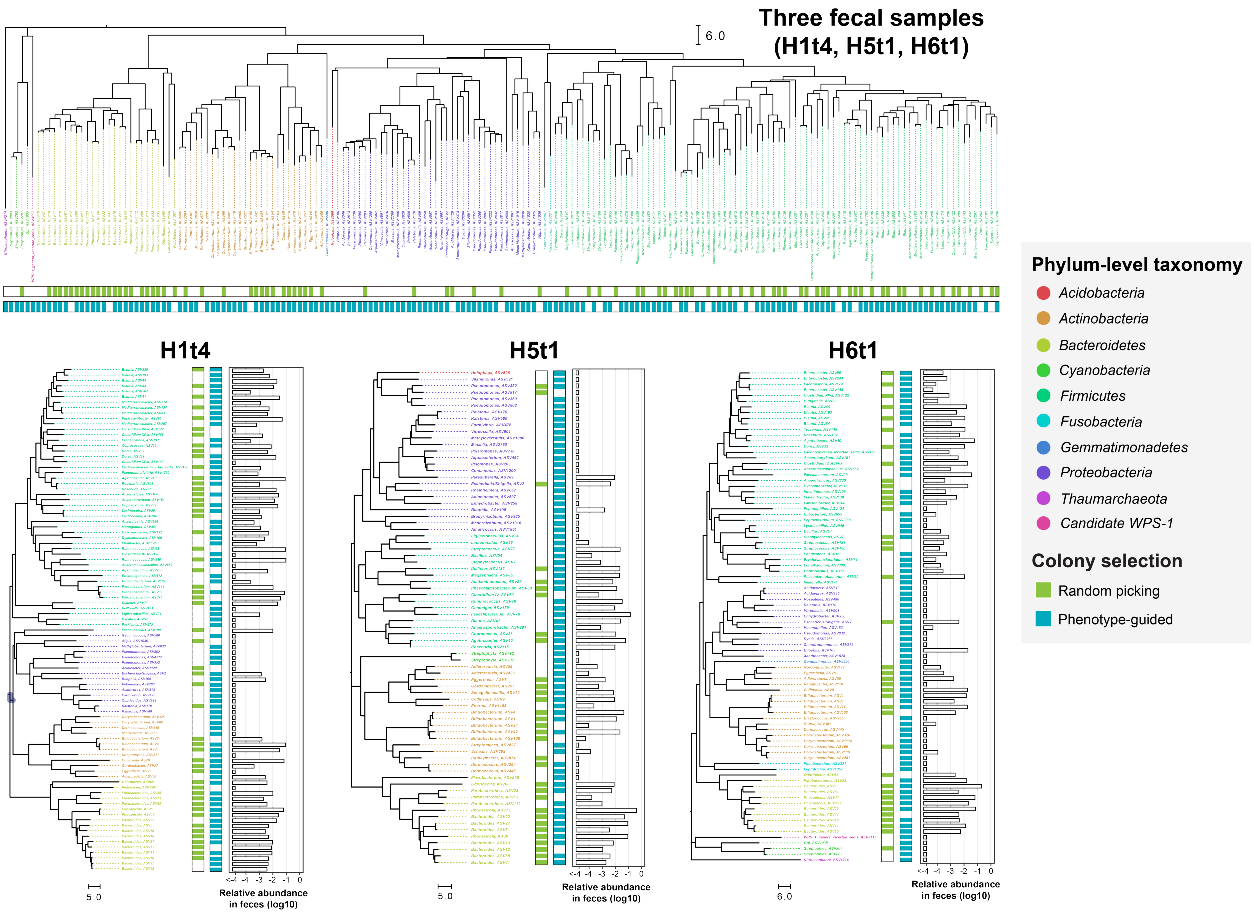

Figure S5: Phylogeny of isolated taxa and their abundances in original feces for three samples. Phylogeny of isolated ASVs from any of three feces samples (top panel), i.e., H1t4, H5t1 and H6t1, or each feces sample separately (bottom panels) based on 16S-V4 sequences. White bar in bottom panels shows the relative abundance in original fecal samples. Branch color distinguishes bacterial phylum, and the colored bars indicate isolates obtained by random picking or by phenotype-guided selection.

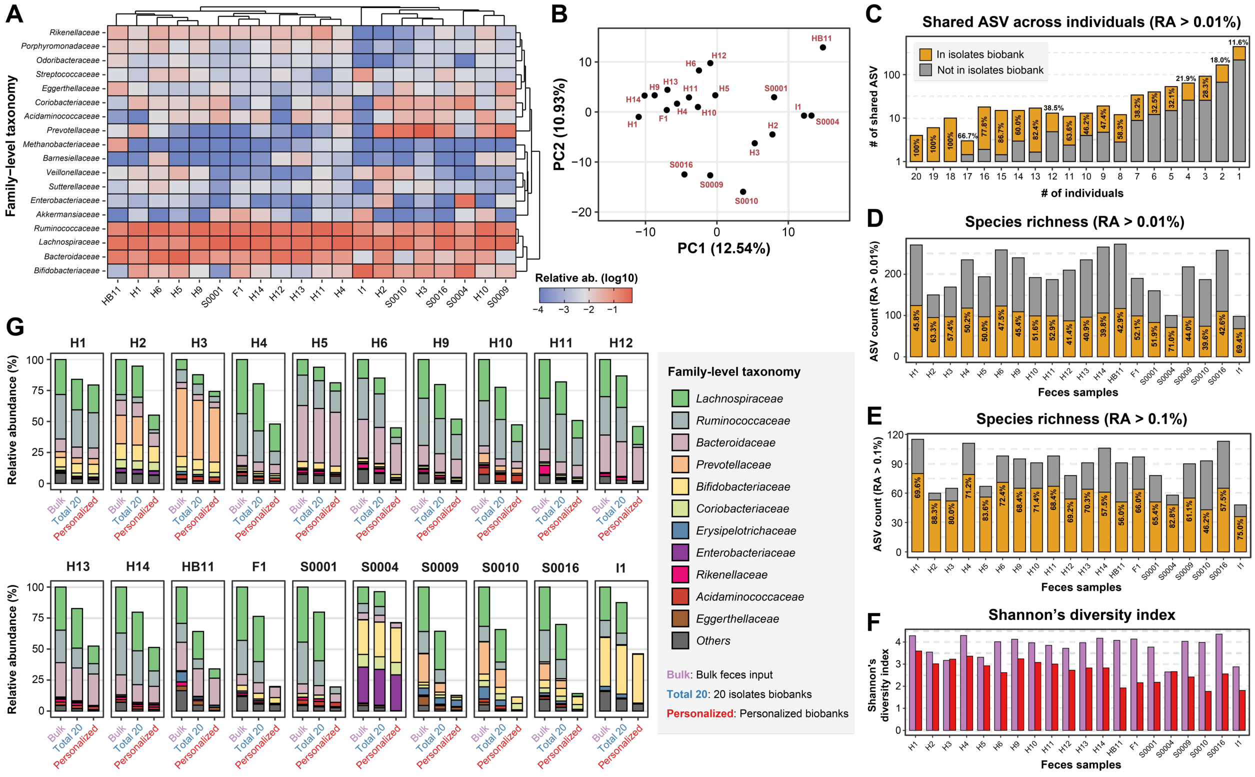

Figure S6: Diversity analysis of 20 personalized isolates collections and gut microbiota in bulk feces samples.

(A) Heatmap of family-level relative abundance for gut microbiota in 20 individuals used in this study. Individuals were classified into different groups by hierarchical clustering, representing their different enterotypes.

(B) PCA ordination of individuals based on their ASV-level gut microbiota profiles.

(C) Bar plot showing most highly prevalent ASVs are isolated in our biobanks. ASVs are classified into different groups based on their prevalence in original feces samples across 20 individuals. Numbers on the bars represent the proportion of ASVs isolated in our biobanks.

(D, E) Species richness, i.e., numbers of ASVs, of original feces samples from 20 individuals. ASVs with average relative abundance > 0.01% **(D)** or 0.1% **(E)** are shown. Numbers on the bars represent the proportion of ASVs isolated in our biobanks.

(F) Comparison of Shannon's diversity index between bulk feces (in purple) and personalized isolates biobanks (in red).

(G) Comparison of abundance profile in original feces of ASVs in bulk feces (in purple), across all biobanks (in blue) and personalized isolates biobanks (in red).

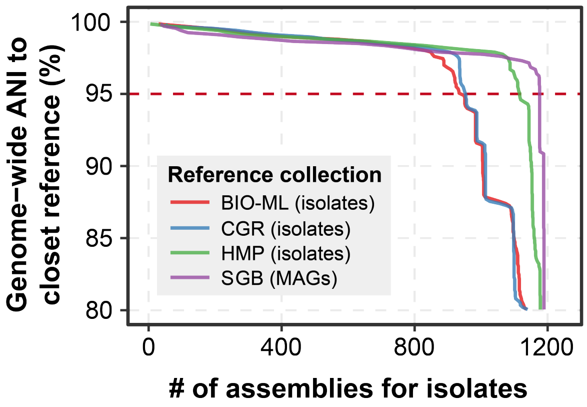

Figure S7. Comparison between gut strains from 20 personalized biobanks and existing gut microbiota database.

Genome-wide average nucleotide identity (ANI) of 1,197 isolates genomes to the closest BIO-ML, CGR, HMP and SGB reference genome collections. The red horizontal line represents 95% ANI.

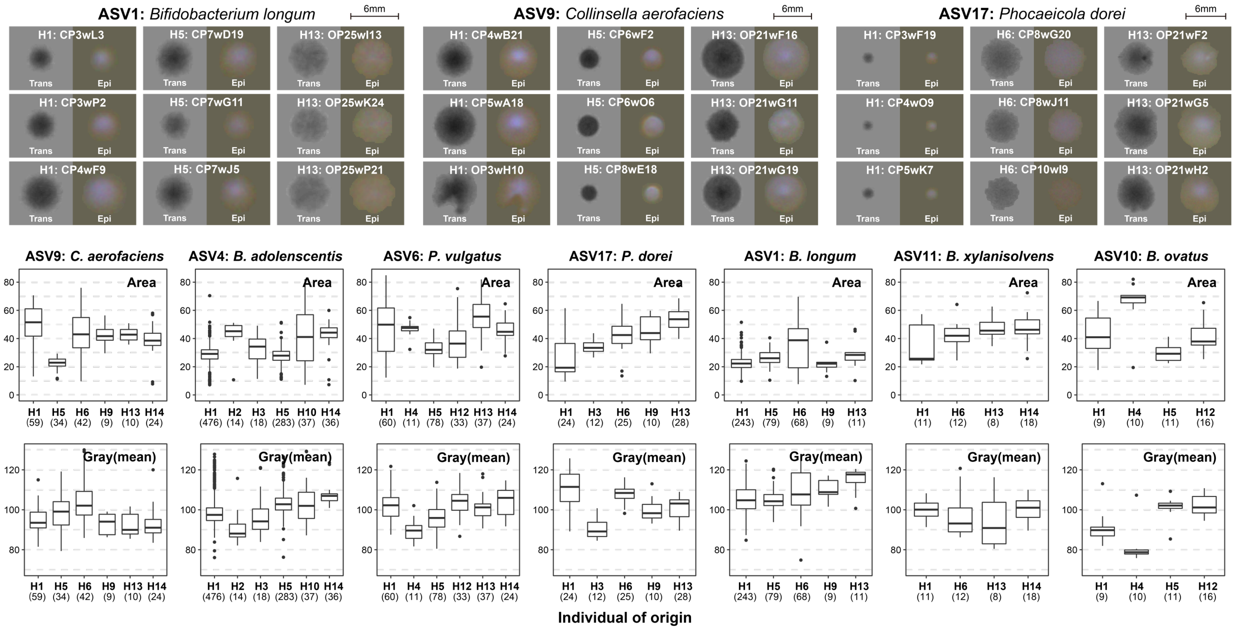

Figure S8: Colony morphologies of isolates from different individuals.

Examples of trans-illuminated and epi-illuminated colony images for three ASVs (*B. longum* ASV-1, *C. aerofaciens* ASV-9 and *P. dorei* ASV-17) from the same individual or different individuals are shown in top panels. Comparison of colony area and density (mean of gray in trans-illuminated images) for more ASVs are shown in bottom panels as box-and-whisker plots. The numbers in the brackets under individuals of origin represent the number of isolates shown in the box plots. Definition of box-plot elements: center line: median; box limits: upper and lower 25th quartiles; whiskers: 1.5x interquartile range.

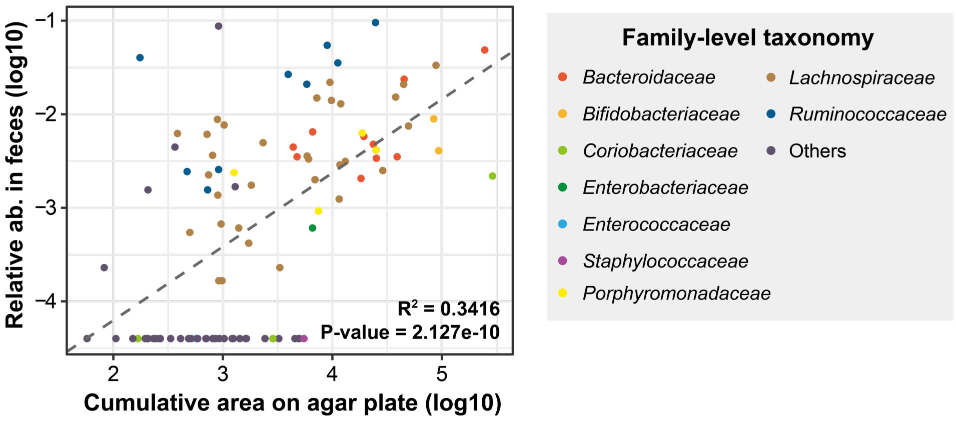

Figure S9: Comparison of bacterial growth on solid agar plates and abundance in original feces. Correlation between cumulative colony area of ASVs and their relative abundance in original fecal sample. ASVs are colored based on family-level taxonomy. The P-value was calculated by two-sided t-test on the linear regression to test if coefficient of the variable (cumulative area on agar plate) equals to zero in the regression.

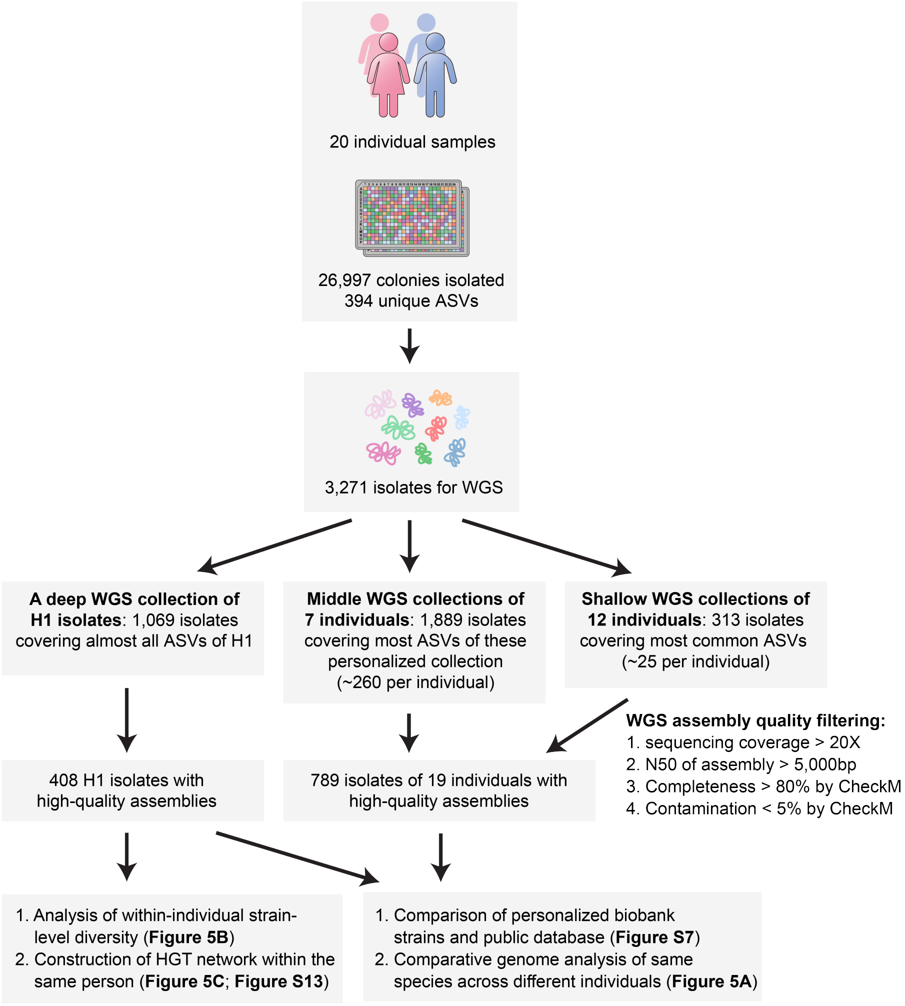

Figure S10: Isolates selected for WGS from 20-person gut microbiota biobanks.

Information of genome assembly quality filtering and WGS datasets used by different analysis in this study.

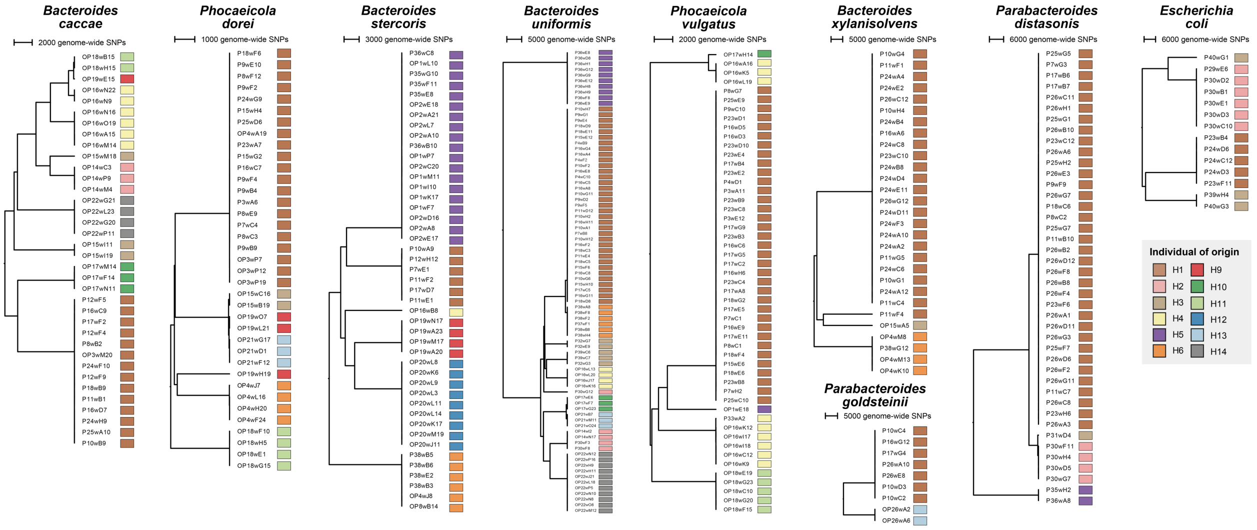

Figure S11: SNP-based phylogeny for isolates of prevalent species in entire collection.

Genome-wide SNP profiles of isolates with high-quality genomes were concatenated and analyzed by MEGAX to construct UPGMA tree for each species. Colored bars represent the individual of origin for isolates.

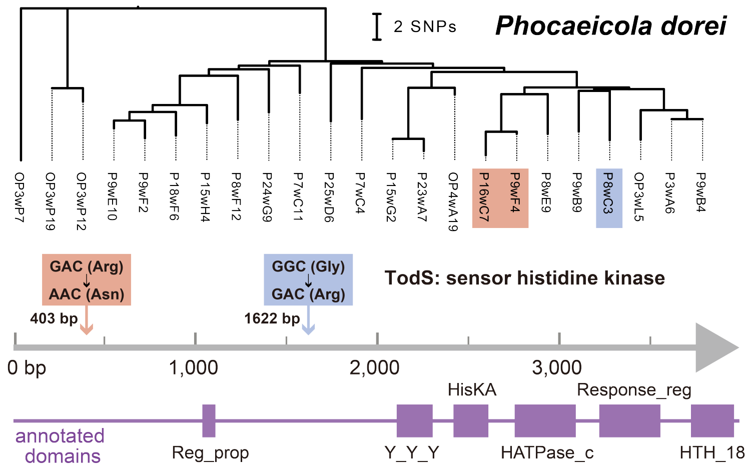

Figure S12: Gene-level convergent adaptive evolution of *P. dorei* in individual H1.

Phylogeny of *P. dorei* isolates from H1 were constructed based on their SNP profiles (top panel). Two lineages with different coding SNP variants in same *todS* genes were highlighted by shade in the phylogenetic tree and locations of SNPs were highlighted on gene annotation (bottom panel).

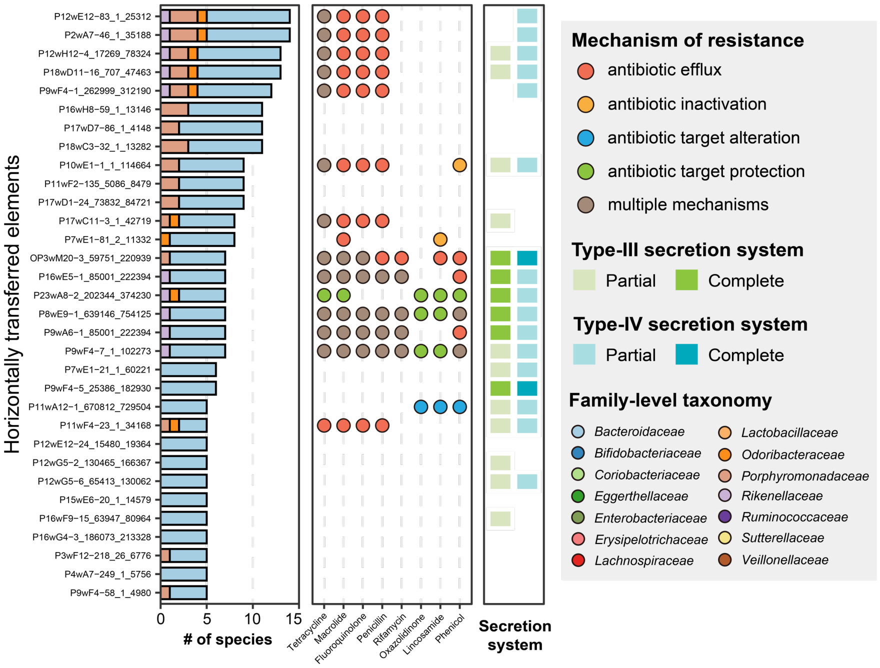

Figure S13: Prevalence and functional annotation of most widespread HGT elements.

Bar-plot on the left shows the prevalence of HGT elements across different species. Heatmap in the middle shows presence of ARGs with different mechanisms of action and heatmap on the right shows the presence of complete or partial Type-III or Type-IV secretion systems.
