# Supplementary material for: High-throughput microbial culturomics using automation and machine learning
Source: Nat Biotechnol. 2023 Feb 20;41(10):1424–33. doi: 10.1038/s41587-023-01674-2 (PMC10567565; doi:10.1038/s41587-023-01674-2)
Supplement: Supplementary file 2 — Reporting Summary [file 41587_2023_1674_MOESM2_ESM.pdf]

nature research

Corresponding author(s): Harris H. Wang

Last updated by author(s): Dec 29, 2022

Reporting Summary

Nature Research wishes to improve the reproducibility of the work that we publish. This form provides structure for consistency and transparency in reporting. For further information on Nature Research policies, see our Editorial Policies and the Editorial Policy Checklist.

Statistics

For all statistical analyses, confirm that the following items are present in the figure legend, table legend, main text, or Methods section.

n/a	Confirmed	
☐	☒	The exact sample size (*n*) for each experimental group/condition, given as a discrete number and unit of measurement
☐	☒	A statement on whether measurements were taken from distinct samples or whether the same sample was measured repeatedly
☐	☒	The statistical test(s) used AND whether they are one- or two-sided<br>*Only common tests should be described solely by name; describe more complex techniques in the Methods section.*
☒	☐	A description of all covariates tested
☐	☒	A description of any assumptions or corrections, such as tests of normality and adjustment for multiple comparisons
☐	☒	A full description of the statistical parameters including central tendency (e.g. means) or other basic estimates (e.g. regression coefficient) AND variation (e.g. standard deviation) or associated estimates of uncertainty (e.g. confidence intervals)
☐	☒	For null hypothesis testing, the test statistic (e.g. *F*, *t*, *r*) with confidence intervals, effect sizes, degrees of freedom and *P* value noted<br>*Give P values as exact values whenever suitable.*
☒	☐	For Bayesian analysis, information on the choice of priors and Markov chain Monte Carlo settings
☒	☐	For hierarchical and complex designs, identification of the appropriate level for tests and full reporting of outcomes
☐	☒	Estimates of effect sizes (e.g. Cohen's *d*, Pearson's *r*), indicating how they were calculated

Our web collection on statistics for biologists contains articles on many of the points above.

Software and code

Policy information about availability of computer code

Data collection	Illumina sequencing data was collected with MiSeq, NextSeq or HiSeq Control Software. Images of plates were acquired with Hudson RapidPick control software of CAMII system.
Data analysis	Scripts used to analyze plate images in this study can be accessed at https://github.com/hym0405/CAMII. Raw reads of 16SV4 amplicon were analyzed by USEARCH v11.0.667; Taxonomy of ASVs were assigned using Ribosomal Database Project classifier v2.13; Multi-sequence alignment was performed on ASV sequences using MUSCLE v5 and analyzed by MEGA v11.0.11 to calculate phylogeny reconstruction; Raw reads of whole-genome sequencing were processed by Cutadapt v2.1 and assembled by Unicycler v0.4.4; The quality and taxonomy of draft genomes were assessed by QUAST v4.6.3, CheckM v1.0.13 and GTDB-Tk v0.2.2; Processed Illumina reads were aligned by Bowtie2 v2.3.4 and alignments were processed by SAMtools v1.9 and BCFtools v1.9 to call genomic variation. The average nucleotide identity (ANI) between genomes were calculated by FastANI v1.0. To identity HGT between isolates, genomes of different species were compared by BLASTN v2.7.1 and sequences of HGT elements were annotated by Prokka v1.12.

For manuscripts utilizing custom algorithms or software that are central to the research but not yet described in published literature, software must be made available to editors and reviewers. We strongly encourage code deposition in a community repository (e.g. GitHub). See the Nature Research guidelines for submitting code & software for further information.

Data

Policy information about availability of data

All manuscripts must include a data availability statement. This statement should provide the following information, where applicable:

- Accession codes, unique identifiers, or web links for publicly available datasets
- A list of figures that have associated raw data
- A description of any restrictions on data availability

Taxonomy of ASVs were assigned based on 16S rRNA training set 18 provided by Ribosomal Database Project. The annotation of ARG genes in HGT elements is based on CARD database v3.1.4 and annotation of Secretion systems was based on EffectiveDB database.

The sequencing data generated in this study have been submitted to the NCBI BioProject database (http://www.ncbi.nlm.nih.gov/bioproject/) under accession number PRJNA745993 and other associated data of the strain collection, including morphological features and raw images, can be accessed at http://microbial-culturomics.com.

Field-specific reporting

Please select the one below that is the best fit for your research. If you are not sure, read the appropriate sections before making your selection.

☒ Life sciences ☐ Behavioural & social sciences ☐ Ecological, evolutionary & environmental sciences

For a reference copy of the document with all sections, see nature.com/documents/nr-reporting-summary-flat.pdf

Life sciences study design

All studies must disclose on these points even when the disclosure is negative.

Sample size	No sample size calculations were performed as the number analyzed depended on the yield from the experiment; sample sizes are listed in Methods section where applicable. The sample sizes in this study are # of colonies imaged or # of isolates collected, and are based on experimental yields. The specific # of colonies or isolates was chosen in this study to ensure they cover majority of morphological diversity or phylogenic diversity of human gut microbiota.
Data exclusions	Data exclusion was based on sequencing coverage or genome quality to remove technical artifacts as described in the Methods section.
Replication	In proof of concept, phenotype-guide strain isolation was performed for samples from 3 individuals as biological replicates (Figure 1F and Figure S4). Bacterial genus prediction based on morphological features were bootstrapped for 20 times as technical replicates (Figure 3D&E). The phenotype-guide strain isolation was also performed for the gut microbiota of remaining 17 individuals in a year with all isolation experiments performing consistently well on these individuals based on the criterion of isolated ASVs (Figure S6).
Randomization	For all isolates generated in this study, individual of origins for isolated gut strains were assigned based on the defined identity of original feces (Table S4) and covariate is not applicable here. For the simulation for in silico isolation and bootstrapping for morphology-based taxonomy prediction, randomized initialization was applied.
Blinding	The image data acquisition was performed by CAMII system automatically during the experiment, thus was blinded to researchers. Automated isolation was performed by CAMII system and the researchers were blinded to isolation strategy in genomic DNA extraction and library preparation as different groups are mixed in the same round of isolation. Blinding in analysis was not possible during experiments as we are comparing isolates between individuals. All analyses of associated data were performed with the same parameters and criteria described in Methods section.

Reporting for specific materials, systems and methods

We require information from authors about some types of materials, experimental systems and methods used in many studies. Here, indicate whether each material, system or method listed is relevant to your study. If you are not sure if a list item applies to your research, read the appropriate section before selecting a response.

Materials & experimental systems

n/a	Involved in the study
☒	☐ Antibodies
☒	☐ Eukaryotic cell lines
☒	☐ Palaeontology and archaeology
☒	☐ Animals and other organisms
☐	☒ Human research participants
☒	☐ Clinical data
☒	☐ Dual use research of concern

Methods

n/a	Involved in the study
☒	☐ ChIP-seq
☒	☐ Flow cytometry
☒	☐ MRI-based neuroimaging

Human research participants

Policy information about studies involving human research participants

Population characteristics	20 healthy human donors as indicated in the text, including 12 males and 8 females with ages mostly ranging from 20 to 50.
Recruitment	20 healthy volunteers were verbally recruited from Columbia University Medical Center. Exclusion criteria were antibiotic exposure in the last 90 days or currently undergoing gastrointestinal disease (self-reported). The donors may be potentially biased to ages of 20 to 50. We anticipated this didn't impact our overall result of isolates collection as well as colony morphological analysis.
Ethics oversight	This study was approved and conducted under Columbia University Medical Center Institutional Review Board protocol AAAR0753. Written informed consent was obtained from the subject in the study.

Note that full information on the approval of the study protocol must also be provided in the manuscript.
